# Comparison of laparoscopic and robotic right hemicolectomy: insights from a single centre

**DOI:** 10.1308/rcsann.2025.0084

**Published:** 2025-11-11

**Authors:** MA Yusufi, U Mateen, M Uneeb, M Gupta, N Muhibullah, NN Siddiqi

**Affiliations:** ^1^ University Hospitals Dorset NHS Foundation Trust, UK; ^2^ Shifa Tameer-e-Millat University, Shifa College of Medicine, Islamabad, Pakistan

**Keywords:** Laparoscopy, Robotic surgical procedures, Colectomy, Colonic neoplasms

## Abstract

**Introduction:**

Right hemicolectomy has evolved from open to minimally invasive approaches. Laparoscopic right hemicolectomy (LRH) is widely adopted, yet challenges persist, particularly in complex dissections. Robotic right hemicolectomy (RRH) offers enhanced precision and ergonomics but remains under scrutiny for its comparative advantages. This study critically evaluates the perioperative outcomes of LRH vs RRH within a single-centre framework.

**Methods:**

A retrospective cohort analysis was carried out at a single tertiary institution, reviewing all RRH cases from January 2021 to December 2024 and comparing them with the most recent LRH cases. Inclusion criteria encompassed adults undergoing elective right hemicolectomy for confirmed malignancy; patients with recurrent disease or emergency indications were excluded. Demographics, tumour characteristics, operative details and short-term outcomes were compared using appropriate statistical tests.

**Results:**

The baseline demographics were comparable between the groups. Transverse colon tumours were more frequent in the RRH group (*n* = 15, 37.5%) than in the LRH group (*n* = 2, 5%) (*p* = 0.003). The mean operative time was comparable in both groups (219.9mi in RRH vs 206.8min in LRH; *p* = 0.277). RRH demonstrated significantly reduced blood loss (24 vs 50.1 m; *p* < 0.001), earlier return of bowel function (1.9 vs 2.6 days; *p* = 0.004), and a reduced hospital stay (3.5 vs 6.1 days; *p* = 0.001). Lymph node yield was comparable.

**Conclusion:**

RRH offers measurable benefits in recovery and haemostasis as compared with LRH. It offers equivalent short-term oncological outcomes without prolonging the operative time. Our findings suggest RRH may be preferable for anatomically complex cases. Larger, prospective studies with a long-term follow-up period are therefore indicated.

## Introduction

Colorectal cancer (CRC) ranks as the third most common type of cancer and the second highest cause of cancer-related death across the globe.^1^ In 2020, the incidence of CRC was around 1.9 million, with more than 930,000 mortalities. These numbers are projected to rise due to demographic transitions and lifestyle shifts.^2,3^ Specifically, right-sided colonic cancers (R-CC) constitute a biologically and clinically distinct subset.^4^ These tumours tend to present with more advanced disease at diagnosis, are frequently associated with microsatellite instability and mucinous histology, and often carry a worse prognosis than their left-sided counterparts.^5^

Compounding the challenge, R-CC cases are often diagnosed in elderly populations with multiple comorbidities, further complicating management and perioperative risk assessment.^6^ The anatomical intricacies of the right colon – characterised by variable vascular supply, proximity to the duodenum and the pancreas, and complex lymphatic drainage – render surgical resection technically demanding.^7^ As such, surgical innovation and precision are critical in achieving optimal oncological clearance while minimising perioperative morbidity.^8^

Surgical intervention is the standard of care for localised CRC.^9^ Over the past couple of decades, there has been a remarkable shift towards minimally invasive surgery (MIS).^10^ Laparoscopic right hemicolectomy (LRH) has gained global traction compared with traditional open surgery, supported by evidence showing a reduced need for postoperative analgesia, reduced lengths of hospital stay and an accelerated functional recovery.^11^ Although beneficial, conventional laparoscopic surgery is constrained by a two-dimensional operative view, limited mobility of instruments, and a steep learning curve, which can pose challenges during intricate lymphovascular dissection and intracorporeal anastomosis.^12^

To overcome these limitations, robotic-assisted surgery has evolved as an innovative technique in colorectal oncology.^13^ The robotic platform offers three-dimensional (3D) high-definition visualisation, enhanced dexterity through wristed instruments, tremor filtration and an ergonomic surgeon console.^14^ These features have shown clear benefits in rectal cancer surgery and are now being extrapolated to right-sided resections.^15,^^16^ However, the clinical and cost-effectiveness of robotic right hemicolectomy (RRH) remain under ongoing investigation, with current literature yielding inconsistent findings regarding operative time, conversion rates, lymph node harvest and overall outcomes.^17^

This single-centre study seeks to conduct a direct, head-to-head comparison between LRH and RRH. By implementing standardised surgical protocols, controlled operative environments and consistent surgical expertise, this study aims to generate high-fidelity data on perioperative metrics, oncological quality and procedural feasibility.^18^ Ultimately, these findings will hopefully contribute to evidence-based optimisation of surgical strategies for R-CC, supporting personalised and technologically integrated care in an era of increasing CRC incidence.

## Methods

A retrospective, non-randomised, comparative cohort study was carried out. Patients meeting the selection criteria were reviewed. The decision for laparoscopic or robotic surgery was primarily based on the availability of the surgical robot (because the gynaecology oncology team uses the same robot). However, there was also some element of surgeon preference, with some surgeons preferring a laparoscopic approach in patients with a higher American Society of Anesthesiologists (ASA) class. Clinical and demographic data were accessed from electronic patient records. Demographic variables such as age, sex, body mass index (BMI), and the ASA Physical Status Classification System (ASA class) were recorded. Tumour characteristics – location, T stage and histology – were included. Perioperative and short-term postoperative outcomes were assessed, including operative time, blood loss, time to first bowel activity, length of hospital stay and nodal harvest. Time to flatus was defined as an indicator of first bowel activity. IBM SPSS Statistics for Windows, version 23.0 (IBM Corp., Armonk, NY, USA) was used for data analysis, using parametric tests such as the independent samples *t*-test for continuous variables and the Pearson’s chi-squared test for categorical variables. A *p*-value less than 0.05 was considered to be statistically significant.

### Operative technique

All operations were performed or supervised by a consultant colorectal surgeon with expertise in both modalities of MIS. For LRH, the patient was placed in the Lloyd-Davies position with shoulder support. The pneumoperitoneum was created with a 12mm umbilical port via the Hasson technique. After assessment of the peritoneal cavity for carcinomatosis, two 5mm ports were inserted in the suprapubic and epigastric region, along with a second 12mm port in the left lower quadrant. The exact location of the ports varied from case to case, depending on the surgeon’s preference, and patient factors such as the body habitus. A standard medial to lateral dissection was completed using a combination of blunt and sharp dissection, with an ultrasonic energy device (Harmonic Scalpel^®^, Ethicon Endo-Surgery, Cincinnati, OH, USA), preserving the duodenum and the head of the pancreas. The ileocolic pedicle was secured with polymer ligating clips (Hem-o-lok^®^ clips, Teleflex Medical, Research Triangle Park, NC, USA). Lateral colonic mobilisation was done. The specimen was exteriorised via a mini-laparotomy either in the midline or the right upper quadrant, depending on the surgeon's preference. A hand-sewn or stapled ileo-transverse extracorporeal anastomosis (ECA) was performed based on surgeon preference. There were no cases of intracorporeal anastomosis (ICA).

For RRH, all procedures were carried out using the DaVinci Xi^®^ dual-console surgical system (Intuitive Surgical, Sunnyvale, CA, USA). Patient positioning was the same as described for LRH. The pneumoperitoneum was created with a Veress needle in the left upper quadrant. Four robotic ports (8mm) were inserted in a straight line at right angles to the target site, 6–8cm apart and about 4cm above the pubic symphysis. A 12mm valveless trocar (AirSeal^®^ access port, CONMED, Utica, NY, USA) was inserted (for the assistant) in the region of the left flank. The operative steps were the same as for LRH. ECA or robotically sutured ICA was performed based on surgeon preference.

## Results

Between January 2021 and December 2024, 40 patients underwent RRH. These were compared with the 40 most recent LRH cases conducted within the same period. This retrospective comparison was designed to evaluate demographic parity and ensure comparability between cohorts prior to assessing outcomes. The overall cohort had a mean age of 73.8 ± 9.4 years (range 37–92), with 28 men (35%). The average BMI was 29.7 ± 6.9kg/m² (range 18.8–62.1). Regarding ASA class, only 1 patient (1.25%) was classified as ASA I, 28 (35%) were classified as ASA II, and the majority – 51 patients (63.75%) – as ASA III.

For statistical analysis, ASA I and II were grouped (ASA I/II) to facilitate comparisons (Table 1). Although not statistically significant (*p* = 0.115), the LRH group had a slightly higher mean age than the RRH group (75.4 ± 9.5 vs 72.1 ± 8.9 years). Gender distribution was similar, with male patients accounting for 32.5% in the LRH group and 37.5% in the RRH group (*p* = 0.815). The mean BMI was also nearly identical between groups (29.8 ± 5.0 in LRH vs 29.6 ± 8.5 in RRH; *p* = 0.867). ASA classification showed a trend toward lower-risk patients in the RRH group, in which 45% were ASA I/II compared with 27.5% in the LRH group. ASA III patients constituted 72.5% of the LRH group and 55% of the RRH group. Although not statistically significant (*p* = 0.163), this suggests a possible selection bias favouring patients with fewer comorbidities for the robotic approach. No cases required a conversion to open surgery in either group. In the LRH group, one (2.5%) patient experienced an anastomotic leak, whereas the RRH group recorded one (2.5%) mortality due to a pulmonary embolism. All resections were R0 resections.

**Table 1:** Patient demographics

Demographic	Total (*N* = 80)	LRH (*N* = 40)	RRH (*N* = 40)	*p*-value
Age (years) (*M* ± sd)	73.8 ± 9.4	75.4 ± 9.5	72.1 ± 8.9	0.115
Male, *n* (%)	28 (35)	13 (32.5)	15 (37.5)	0.815
BMI (kg/m²) (*M* ± sd)	29.7 ± 6.9	29.8 ± 5.0	29.6 ± 8.5	0.867
ASA I/II, *n* (%)	29 (36.25)	11 (27.5)	18 (45)	0.163
ASA III, *n* (%)	51 (63.75)	29 (72.5)	22 (55)	

ASA = American Society of Anesthesiologists physical status classification system; BMI = body mass index; LRH = laparoscopic right hemicolectomy; *M* = mean; RRH = robotic right hemicolectomy; SD = standard deviation

**Figure 1:**
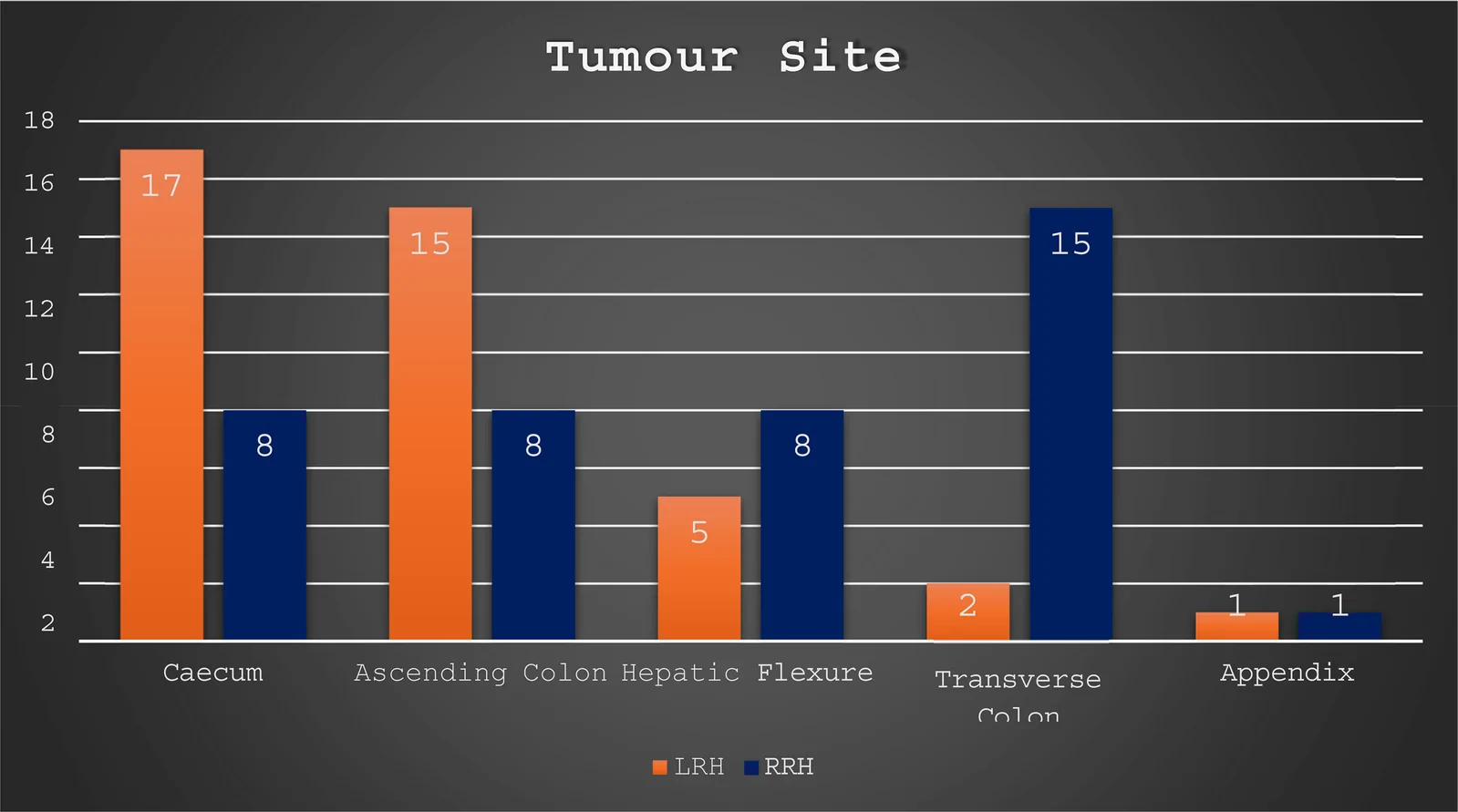
Tumour site

**Figure 2:**
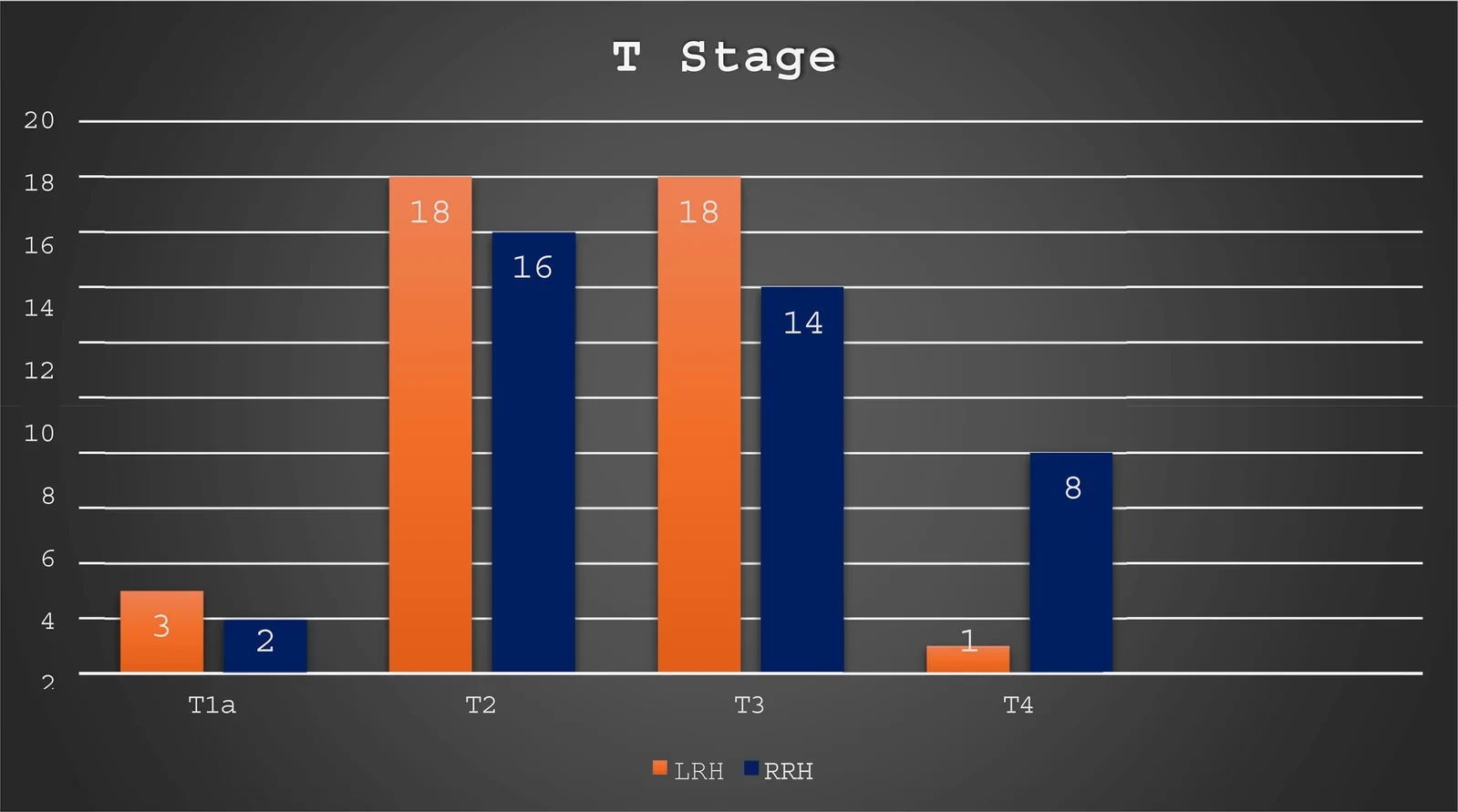
T stage

**Figure 3:**
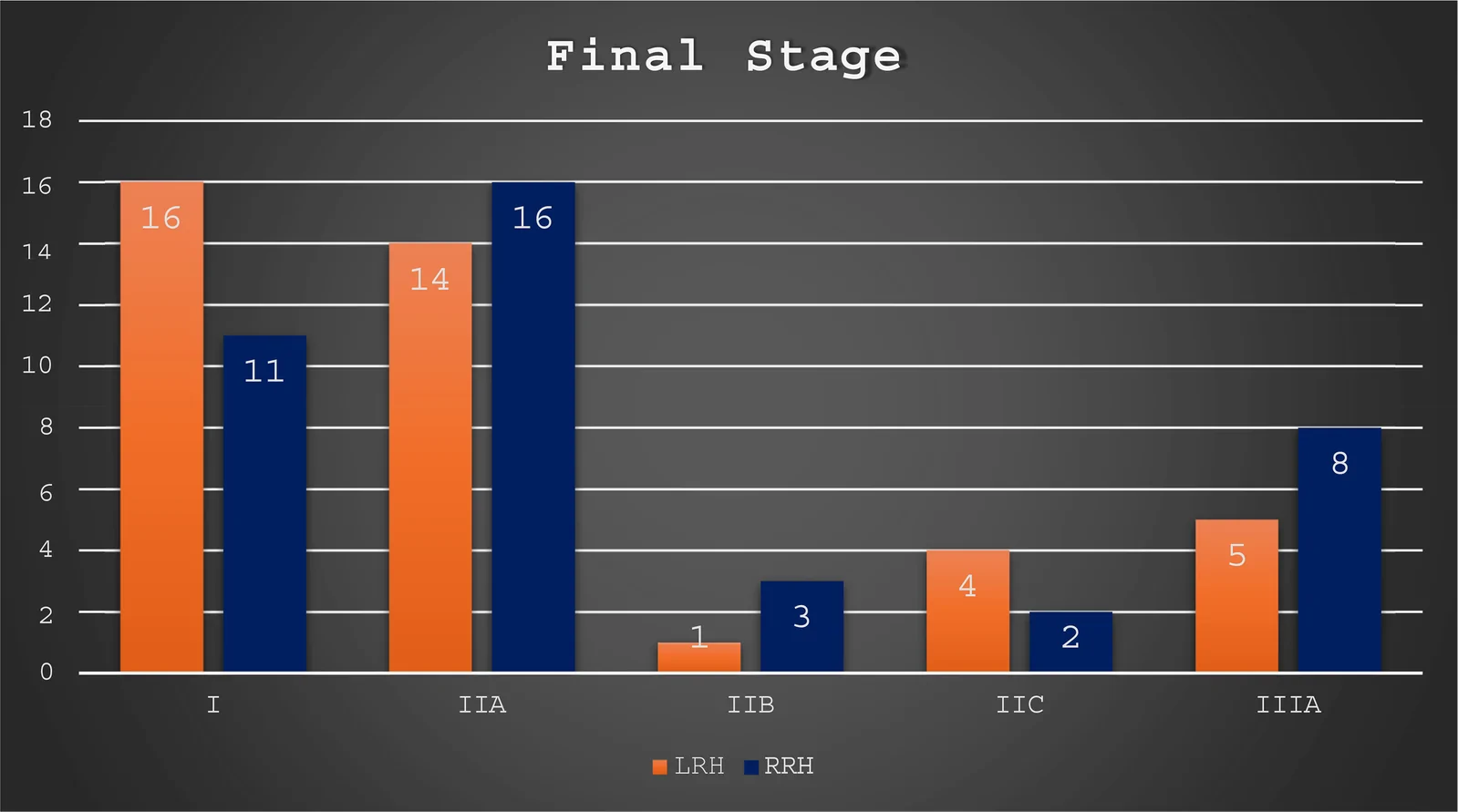
Final histological stage

Tumour characteristics, including anatomical location, T stage and overall cancer staging per American Joint Committee on Cancer (AJCC) eighth edition criteria, are presented in Figures 1–3. These parameters were assessed to identify any confounding oncological disparities between groups that could affect operative or oncological outcomes. Tumour location varied between groups: LRH had more caecal and ascending colonic cancers, whereas RRH had more proximal transverse colonic tumours (*p* = 0.003), as shown in Figure 1.

In summary, both groups were statistically comparable across key baseline variables, although the RRH group included a higher proportion of lower-risk patients. This imbalance, although not reaching statistical significance, should be considered when interpreting downstream clinical outcomes.

Table 2 compares outcomes in LRH with RRH. The factors measured include operative time, blood loss, time to first bowel activity (indicated by first flatus), overall hospital stay and nodal harvest. Both procedures were similar with regard to operative time and nodal harvest.

**Table 2:** Outcomes comparison between laparoscopic right hemicolectomy (LRH) and robotic right hemicolectomy (RRH)

Outcomes	LRH	RRH	*p*-value
Operative time (min) (M ± sd) (range)	206.8 ± 51.3 (90–360)	219.9 ± 55.8 (130–395)	0.277
Blood loss (ml) (*M* ± sd) (range)	50.1 ± 26.0 (5–150)	24.0 ± 19.6 (5–100)	**<0.001**
Time to bowel activity (days) (*M* ± sd) (range)	2.6 ± 1.0 (1–5)	1.9 ± 1.0 (1–5)	**0**.**004**
Hospital stay (days) (*M* ± sd) (range)	6.1 ± 4.7 (1–30)	3.5 ± 1.7 (2–10)	**0**.**001**
Nodal harvest (*n*) (*M* ± sd) (range)	24.2 ± 9.0 (7–55)	26.6 ± 6.7 (15–47)	0.175

M = mean; sd = standard deviation

## Discussion

This single-centre retrospective comparison of LRH and RRH offers critical insights into contemporary colorectal surgical practice. The findings affirm certain anticipated advantages of robotic approaches and highlight areas where parity exists, thus enriching the discourse around the optimal choice for minimally invasive right hemicolectomy.

### Patient demographics and baseline characteristics

Our study demonstrated comparability in baseline demographics between the LRH and RRH groups. The mean age, gender distribution, BMI and ASA classifications showed no statistically significant differences. The mean age was 75.4 ± 9.5 years in the LRH group, whereas it was 72.1 ± 8.9 years in the RRH group (*p* = 0.115). Similarly, BMI and gender distribution were matched, underscoring that the cohort selection did not introduce bias based on these parameters. The nonsignificant difference in ASA classifications (*p* = 0.163) also supports comparable baseline operative risks between the two groups.

This parity is essential when evaluating surgical outcomes, ensuring that observed differences stem from the surgical approach rather than patient characteristics. Moreover, it aligns with earlier studies such as those by Spinoglio *et al* and Park *et al*, which emphasise the importance of matching demographic profiles to reduce confounding.^19,20^ Spinoglio *et al* conducted a study on 202 patients undergoing right hemicolectomy with complete mesocolic excision. This study demonstrated a lower conversion rate (0% vs 6.9%, *p* = 0.01) but lengthier surgeries (279 vs 236min, *p* < 0.001) for robotic surgery as opposed to standard laparoscopy.

### Tumour characteristics

A notable difference was observed in the location of the tumour. LRH predominantly involved caecal and ascending colon cancers, whereas RRH was more frequently used for proximal transverse colon tumours (*p* = 0.003). This may be due to a subconscious selection bias, given the increased technical demands and limited working angles in the laparoscopic approach for tumours involving the transverse colon. The robotic platform, by virtue of its enhanced articulation and three-dimensional visualisation, offers ergonomic advantages for dissection in anatomically challenging zones such as the transverse colon. A recent meta-analysis by Trastulli *et al*, analysing 4,148 cases across 12 studies, concluded that RRH was associated with significantly lower intraoperative blood loss (mean difference 16.82ml, *p* < 0.00001), lower postoperative morbidity (odds ratio 0.74, *p* = 0.02) and fewer surgical site infections (risk difference −0.02, *p* = 0.03) compared with LRH. Despite a higher operative time (mean difference 41.52min, *p* < 0.00001) and higher cost, RRH also showed faster recovery, with a shorter time to first flatus (*p* = 0.003) and a reduced hospital stay (*p* = 0.01). These findings favour the use of robotic surgery in technically demanding resections, including those involving the transverse colon.^21^

### Operative time

The operative time was similar in both groups (219.9 ± 55.8min in RRH vs 206.8 ± 51.3min in LRH; *p* = 0.277). This aligns with de’Angelis *et al*, who found similar mean operative times in both groups (*p =* 0.408), confirming no significant time savings after adjusting for the learning curve.^22^ The lack of significant time difference in our cohort may reflect the procedural maturation and enhanced workflow at our centre, particularly given the consistent team involved in both robotic and laparoscopic surgeries. Furthermore, previous studies have demonstrated that the time differential becomes negligible once the robotic learning curve is surpassed.^23^

### Intraoperative blood loss

A significant reduction in intraoperative blood loss was noted in the RRH group (24.0 ± 19.6ml) compared with the LRH group (50.1 ± 26.0ml; *p* < 0.001). Lim *et al* reported similar findings favouring RRH with a mean difference of –19.49ml (95% confidence interval –27.10 to –11.89, *p* < 0.0001) favouring RRH. Spinoglio *et al* similarly noted lower blood loss in robotic-assisted colorectal surgeries. These consistent findings underscore the haemostatic advantage of robotic systems, particularly in anatomically complex procedures, contributing to improved perioperative safety in colonic cancer resections.^24,25^ Robotic surgery facilitates meticulous dissection and precision vascular control through tremor filtration, articulated instrumentation and enhanced visual feedback. These attributes are particularly valuable during central vascular ligation, contributing to improved haemostasis and decreased transfusion requirements. Although the clinical relevance of reduced blood loss in typically low-volume procedures, such as right hemicolectomy, may be modest, the consistency of this finding across the literature supports it as a legitimate advantage.^26^

### Return of bowel function

Time to first bowel activity, as indicated by the first flatus, was significantly shorter in the RRH group (1.9 ± 1.0 days) than the LRH group (2.56 ± 1.0 days), with a *p*-value of 0.004. Several hypotheses can explain this observation. The reduced bowel manipulation in robotic procedures, attributable to better traction and counter-traction mechanics, may attenuate postoperative ileus. Moreover, a more precise mesocolic dissection could mitigate the inflammatory response, contributing to earlier bowel recovery. This outcome aligns with findings from Baik *et al* and a systematic review by Prete *et al*, which link robotic colorectal surgery to a faster return of gastrointestinal function.^27,28^

### Length of hospital stay

Hospital stay was almost halved in the RRH group (3.5 ± 1.7 days) compared with the LRH group (6.1 ± 4.7 days), with a *p*-value of 0.001. This difference is clinically meaningful and suggests a tangible benefit in favour of robotic surgery. These findings align with those of Milone *et al*, who reported significantly shorter hospital stays in RRH patients, even after adjusting for age and comorbidities.^29^ However, hospital stay can also be influenced by institutional discharge policies and patient expectations. Therefore, although encouraging, these findings should be interpreted in context. Furthermore, the economic implications of shorter hospitalisation may offset the higher procedural cost of robotic surgery. A meta-analysis by Chok *et al* analysed 22 randomised controlled trials involving 4,239 patients, comparing open, laparoscopic and robotic colorectal surgery, and found that robotic colorectal surgery resulted in the shortest hospital stays and the lowest mortality rates.^30^

### Nodal harvest

The nodal harvest did not differ significantly between the groups (24.2 ± 9.0 vs 26.6 ± 6.7, *p* = 0.175). Both values exceed the threshold of 12 lymph nodes recommended by oncological guidelines (AJCC eighth edition), indicating oncological adequacy in both techniques. This observation confirms findings from previous studies. A recent meta-analysis comparing robotic-assisted colorectal surgery and laparoscopic colorectal surgery found no significant difference in the number of resected lymph nodes for oncological colorectal surgery.^31^ The robotic platform, although theoretically advantageous for precision dissection, may not offer a distinct benefit in node retrieval due to anatomical limitations rather than technical ones.

### Oncological stage and final histopathology

The distribution of T stages and final histology was comparable across groups. This parity ensures that the surgical approaches were tested against similar oncological challenges. The absence of stage migration or selection bias in the tumour stage affirms the internal validity of our findings.

Although no significant difference in margin status or lymphovascular invasion rates was found, these parameters should be included in future analyses to offer a complete assessment of oncological outcomes.

### Interpretation and contextualisation with existing literature

Our findings resonate with a growing number of recent studies affirming the advantages of RRH in elective right-sided colonic resections. Importantly, the advantages observed in blood loss, time to bowel activity and hospital stay are consistent with high-quality meta-analyses and multicentre trials.^13,32–35^ Nonetheless, it is crucial to temper enthusiasm with pragmatism. The lack of significant differences in operative time and nodal yield reinforces that robotic platforms should not be presumed superior in all facets. Moreover, the learning curve, cost implications and access disparities associated with robotic systems must be acknowledged.^36^ Our centre’s experience underscores that with experienced surgical teams and institutional support, RRH can offer meaningful clinical benefits in select patient populations. However, its implementation must be judicious, guided by surgical complexity, patient comorbidity and availability of resources.

### Limitations and future directions

Although our single-centre study design ensures procedural consistency, it may limit external validity. Future studies should adopt prospective, multicentre designs with a larger sample size to mitigate institutional variability. In addition, long-term oncological outcomes and patient-reported quality-of-life metrics should be integrated to inform surgical decision making more comprehensively. Another limiting factor in our study was the lack of randomisation, as the decision for a laparoscopic or robotic approach was based on surgeon preference and logistics (availability of the robot).

RRH and LRH are broadly considered oncologically equivalent; however, subtle distinctions in pathological outcomes may reflect underlying mechanistic advantages inherent to the robotic platform. One such consideration is the quality of mesocolic excision and central vascular ligation, both of which are pivotal to oncological adequacy. Complete mesocolic excision, as described by Hohenberger *et al*, involves sharp dissection along embryological planes with high vascular ties, preserving mesocolic integrity and maximising lymphadenectomy.^37^ The robotic system, with its stable, magnified 3D visualisation and EndoWrist articulation, potentially enhances adherence to these principles, particularly in anatomically challenging regions such as the transverse colon or at high vascular pedicles near the superior mesenteric vessels.^13,20,38,39^ In our series, the nodal yield was oncologically adequate in both groups; however, the higher mean node count in RRH (although not statistically significant) suggests a potential for more precise lymphovascular dissection. This finding aligns with studies by Shibasaki *et al* and Jarrett *et al*, who noted improved nodal harvest and more consistent D3 dissection in robotic colectomies.^40,41^ Although the clinical impact of higher nodal yield remains debated beyond the recommended minimum, it can enhance staging accuracy, particularly in T3–T4 disease, and possibly influence adjuvant therapy decisions.

Moreover, the reduced intraoperative blood loss observed in the RRH group may not merely reflect improved haemostasis, but could also influence the integrity of tissue planes, inflammatory response and thus postoperative healing. Subclinical mesenteric trauma – often underestimated – can affect lymphatic drainage and immunological surveillance, factors not readily quantifiable but possibly relevant in local recurrence.^42^

## Conclusion

This single-centre retrospective analysis comparing LRH and RRH provides essential insights into the evolving landscape of minimally invasive colorectal surgery. Although both approaches yielded similar lymph node harvests and operative times, RRH demonstrated superior perioperative outcomes, including reduced intraoperative blood loss, faster bowel recovery and shorter hospital stays. These benefits suggest a potential role for RRH in enhancing recovery and minimising surgical stress, particularly in anatomically complex cases, such as those involving transverse colon tumours. However, the study’s methodological limitations – including the lack of randomisation and its retrospective nature, a modest sample size and a short-term follow-up – hinder definitive conclusions. Despite these shortcomings, this study supports the growing evidence that RRH may offer tangible short-term advantages over traditional laparoscopy. Future large-scale, prospective and multicentre studies incorporating comprehensive outcome measures and robust statistical control are essential to validate these findings and guide evidence-based surgical decision making in right-sided colorectal cancer.

## Ethical approval

This is an observational study; therefore, the University Hospitals Dorset Ethics Committee has confirmed that no ethical approval is required for this type of study.

## Consent to participate

Informed consent was waived as the study was observational and involved no intervention or identifiable patient data.

## Author contributions

MA Yusufi: Conceptualisation, Formal analysis, Investigation, Methodology, Project administration, Writing – original draft, Writing – review & editing. U Mateen: Data curation, Methodology, Writing – review & editing. M Uneeb: Conceptualisation, Formal analysis, Methodology, Resources, Software, Writing – review & editing. M Gupta: Data curation, Project administration, Software, Supervision, Visualisation, Writing – review & editing. N Muhibullah: Conceptualisation, Data curation, Formal analysis, Visualisation, Writing – original draft. NN Siddiqi: Conceptualisation, Formal analysis, Investigation, Methodology, Project administration, Supervision, Validation, Visualisation, Writing – review & editing.

## Artificial intelligence

The authors declare that no AI was used to conduct the study or prepare the manuscript.
